# Effects of abiotic factors on ecosystem health of Taihu Lake, China based on eco-exergy theory

**DOI:** 10.1038/srep42872

**Published:** 2017-02-21

**Authors:** Ce Wang, Jun Bi, Brian D. Fath

**Affiliations:** 1 State Key Laboratory of Pollution Control and Resource Reuse, School of the Environment, Nanjing University, Nanjing, 210023, P.R. China; 2 Biology Department, Towson University, Towson, MD 21252, USA; 3 Advanced Systems Analysis Program, International Institute for Applied Systems Analysis (IIASA), Laxenburg, Austria

## Abstract

A lake ecosystem is continuously exposed to environmental stressors with non-linear interrelationships between abiotic factors and aquatic organisms. Ecosystem health depicts the capacity of system to respond to external perturbations and still maintain structure and function. In this study, we explored the effects of abiotic factors on ecosystem health of Taihu Lake in 2013, China from a system-level perspective. Spatiotemporal heterogeneities of eco-exergy and specific eco-exergy served as thermodynamic indicators to represent ecosystem health in the lake. The results showed the plankton community appeared more energetic in May, and relatively healthy in Gonghu Bay with both higher eco-exergy and specific eco-exergy; a eutrophic state was likely discovered in Zhushan Bay with higher eco-exergy but lower specific eco-exergy. Gradient Boosting Machine (GBM) approach was used to explain the non-linear relationships between two indicators and abiotic factors. This analysis revealed water temperature, inorganic nutrients, and total suspended solids greatly contributed to the two indicators that increased. However, pH rise driven by inorganic carbon played an important role in undermining ecosystem health, particularly when pH was higher than 8.2. This implies that climate change with rising CO_2_ concentrations has the potential to aggravate eutrophication in Taihu Lake where high nutrient loads are maintained.

Water quality management can best be upgraded to aquatic ecosystem health assessment by evaluating multiple environmental stressors that can potentially impair ecosystem structure and function and further undermine ecosystem goods and services. Plankton communities play a significant role in establishing aquatic ecosystems, of which the primary producer, phytoplankton, initiates energy flow and chemical cycling driven by solar radiation, and the primary consumer, zooplankton, largely feeds on phytoplankton, in general, with an inverse relationship between their densities because of predator-prey interaction^1^,^2^. Together, they constitute the food source for many aquatic organisms in higher trophic levels, e.g., fish species. Particularly, for eutrophication management in shallow lakes under external stress, a hysteresis phenomenon is observed in the plankton community such that the system remains on the same state until a catastrophic bifurcation is reached at which it shifts to the alternative state^3^,^4^. Catastrophic regime shift has drawn more attention since it can result in loss of species, functions, ecosystem services, ecological, and economic resources and might fundamentally degrade aquatic ecosystem health^5^. Therefore, it is important to apply an integrated system-level indicator to assess the variation trend of lake ecosystem health imposed by external forcing functions^6^,^7^,^8^,^9^.

Exergy, a thermodynamic concept, represents the potential for a given amount of energy to perform work to bring the system to thermodynamic equilibrium^10^,^11^. Studies of ecological systems also include an exergy weighting factor, β, to account for the information embedded in the species genetic complexity^12^. The species-specific β-value is the work energy of an organism including the information relative to the work energy (exergy) with only the biomass chemical exergy. Hence, an organism without information is considered as a fuel which cannot display direct life processes^13^. An ecosystem can utilize input of eco-exergy to increase biomass and further move away from thermodynamic equilibrium by ecological network development and information increase^14^. Eco-exergy could be considered as a holistic ecological indicator of ecosystem development, health and also ecosystem services^15^,^16^, e.g., tropical rainforest (3200 kEURO/ha per year) presented more valuable than desert (20.7 kEURO/ha per year) based on eco-exergy calculation. Specific eco-exergy (also called structural eco-exergy) reflecting the ability of an ecosystem to utilize available resources is defined as the ratio of eco-exergy to total biomass. It gives us the composition of the ecosystem (the more developed organisms the higher specific eco-exergy) and therefore it is a good indicator for ecosystem health assessment, particularly when we focus on lake eutrophication^17^. Therefore, we supplement the use of eco-exergy with specific eco-exergy indictors to assess lake ecosystem health under external stress.

It is very difficult to elucidate the detailed mechanisms on how a lake ecosystem responds to changing environmental and anthropogenic pressures because of the ecosystem complexity that includes nonlinear interactions and heterogeneity among components^18^,^19^,^20^. Therefore, we need a simple and effective mathematical approach to determine ecosystem health changes resulting from external perturbations. Gradient Boosting Machine (GBM) is a decision-tree based approach coupling with a gradient boost algorithm to make accurate estimations of the response variable among highly non-linear relationships in the system of interest, and the principal of the algorithm is to establish new base-learners to be maximally correlated with the negative gradient of the loss function^21^,^22^. Meanwhile, the method can also illustrate the marginal effect of the selected independent variable on the response variable while all other covariates were kept constant at their observations using a partial dependence plot^23^. This kind of modeling has been successfully applied into ecology and biology for simulating non-linear interactions between response and predictor variables^24^,^25^,^26^,^27^. E.g., Randall and Van Woesik 28 investigated the effect of eight sea surface temperature metrics on white-band disease of reef-building corals in the Caribbean, indicating that the disease was associated with climate change, which resulted in the regional population decline. Based on the complexity among components in a lake, GBM can improve the prediction of ecosystem health by combining information from many abiotic factors that individually may not be significant but together are very influential. Using GBM analysis we aim to examine the influence of abiotic factors, e.g., nutrients, temperature, and solar radiation, on the eco-exergy densities of the plankton community to obtain insights into how the ecosystem health of a shallow lake varies.

In this study, we aim to calculate eco-exergy and specific eco-exergy indicators of plankton community at 33 sampling sites in Taihu Lake using monthly-measured phytoplankton and zooplankton biomass during 2013 to illustrate spatiotemporal variation of health level and plankton community dynamics, then apply GBM to determine the effects of abiotic factors on lake ecosystem health. The quantitative relationship between ecological indicators and environmental parameters will help us to predict ecosystem health changes in a shallow lake.

## Material and Methods

### Study site

**Figure 1:**
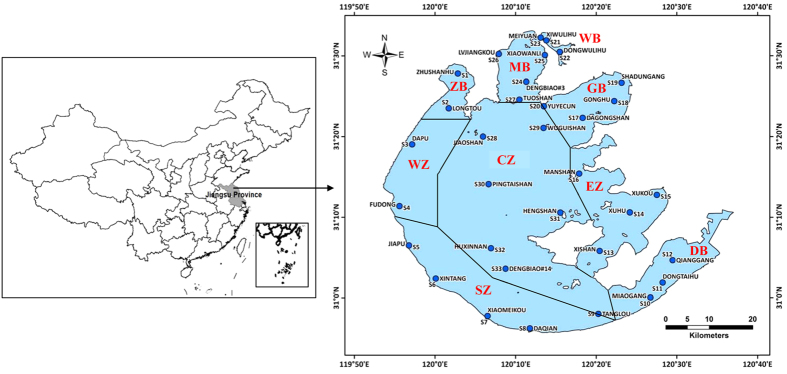
The geographic location of Taihu Lake and sampling sites (The lake is divided into 9 sub-regions - ZB: Zhushan Bay (68.3 Km^2^); WZ: West Zone (199.8 Km^2^); SZ: South Zone (363 Km^2^); DB: Dongtaihu Bay (172.4 Km^2^); EZ: East Zone (268 Km^2^); GB: Gonghu Bay (163.8 Km^2^); WB: Wulihu Bay (5.8 Km^2^); MB: Meilianghu Bay (124 Km^2^); CZ: Center Zone (972.9 Km^2^)). The figure is created by ArcGIS 10.2 (http://www.esri.com).

Taihu Lake located between 30°56′-31°33′N and 119°53′-120°36′E is the third largest freshwater lake in China (see Fig. 1). The shallow lake has a surface area of approximately 2338 Km^2^ and average depth of 1.9 m^29^. It provides various ecosystem services such as agriculture, fishing, tourism, navigation, etc., and also receives considerable point-source and non-point source pollution from the surrounding watershed^30^,^31^. In general, the Taihu Lake basin experiences a typical subtropical monsoon climate with four distinct seasons: spring (March, April, May), summer (June, July, August), fall (September, October, November) and winter (December, January, February). The lake has been in an oligotrophic state since the 1950 s and subjected to a rapid water quality deterioration during the 1980 s, and further suffered from advanced eutrophication since the 1990s^32^,^33^, and it appeared to be hypertrophic state in the late 1990s^34^. A notorious outbreak of non-N_2_-fixing cyanobacteria *Microcystis* occurred in May 2007 which resulted in more than two million citizens without drinking water for a week^35^,^36^. This drew much attention from local authorities and great efforts to manage the lake eutrophication have been made. At the present time, the lake remains in a eutrophied state with aquatic plants present around Dongtaihu Bay.

### Sample collection

Field measurements of the lake ecosystem were regularly carried out at monthly intervals in 2013 at 33 sampling locations in Taihu Lake, as shown in Fig. 1. The 33 sampling locations belong to nine sub-regions, namely Zhushan Bay (ZB, two stations), West Zone (WZ, two stations), South Zone (SZ, five stations), Dongtaihu Bay (DB, three stations), East Zone (EZ, four stations), Gonghu Bay (GB, four stations), Wulihu Bay (WB, three stations), Meilianghu Bay (MB, four stations) and Center Zone (CZ, six stations). On the first ten-day period of each month, we collected water samples at a depth of 0.5 m at each site by driving a vessel with the help of an instrument of navigation-GPS. For each field investigation, approximately 10–12 sampling locations could be visited.

Ammonia nitrogen (NH_4_-N), nitrite nitrogen (NO_2_-N), nitrate nitrogen (NO_3_-N), total nitrogen (TN), orthophosphate (PO_4_-P), dissolved total phosphorous (DTP), total phosphorous (TP), Chlorophyll-a (Chla) and total suspend solids (TSS) concentrations were determined by laboratory analysis within 24 h after sampling. Dissolved oxygen (DO), water temperature (WTEMP), pH, secchi disc depth (SDD) and wind speed (WIND) were directly measured on the spot using a portable monitoring device. Water depth (WD) at each site was calculated by subtracting lake bed elevation from the lake water level. During 2013, in the whole lake, we identified a total of 139 species of phytoplankton (Phyt), of which the dominant species were *Chlorophyta, Bacillariophyta* and *Cyanophyta*; meanwhile, 102 species of zooplankton (Zoop) were observed, of which the *protozoa* were the most abundant in number count while *cladocera* and *copepoda* had the largest biomass. All of phytoplankton and zooplankton biomass were measured in wet weight. Meteorological data of precipitation (PREC) in Wuxi city and solar radiation (SOLR) in Nanjing city stations were obtained from the China Meteorological Data Sharing Service System of the National Meteorological Information Center. Due to constrained resources, PREC and SOLR observations in time series from respective meteorological station were assumed to be representative of the whole lake, while all other water quality, ecological and meteorological data were determined synchronously for each sampling time and site. The detailed information on water sampling, sample pretreatment and laboratory analysis with defined methods for environmental protection standards of China were listed in Supplement Material, Section 1 and Section 2.

### Eco-exergy and specific eco-exergy estimates of plankton community

To calculate eco-exergy, we multiplied the biomass concentration of an organism in the plankton community by the corresponding β-value^37^,^38^. Empirical conversion factors were used to convert wet weight to dry weight biomass in carbon unit (also see Supplementary Material). Using the C-biomass concentrations, we calculated the eco-exergy density measuring per volume eco-exergy. At each sampling date and site, we summed all eco-exergy densities of phytoplankton and zooplankton species to obtain an overall index for the plankton community (see Equation (1)). Then, specific eco-exergy was expressed by eco-exergy per unit of total C-biomass of plankton community (see Equation (2)).


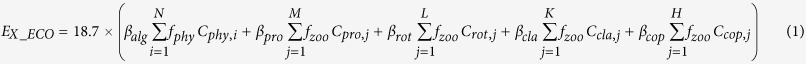



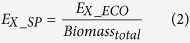


where 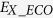 [J/L] is eco-exergy density expressed in detritus equivalent; *β*_*alg*_, *β*_*pro*_, *β*_*rot*_, *β*_*cla*_ and *β*_*cop*_ [dimensionless] are 20, 39, 163, 232 and 232 for planktonic algae, zooplankton - protozoa, rotifers, cladocera and copepoda, respectively^12^. *C*_*phy, i*_, *C*_*pro, j*_, *C*_*rot, j*_, *C*_*cla, j*_ and *C*_*cop, j*_ [mg/L] represent the wet weight biomass of the *i*th phytoplankton species and the *j*th zooplankton species, respectively. *f*_*phy*_ and *f*_*zoo*_ are 0.16 and 0.06 served as the conversion factors of wet weight to C-biomass for phytoplankton and zooplankton species, respectively^39^,^40^,^41^,^42^; The multiplier factor of 18.7 kJ/g changes 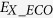 into the energy expressed as organic matter equivalent energy, because the average eco-exergy of detritus is 18.7 kJ/g. *N, M, L, K* and *H* are equal to the number of phytoplankton and zooplankton species, respectively. 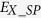 [kJ/g] is specific eco-exergy and 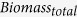 [mg/L] is the sum of phytoplankton and zooplankton C-biomass in dry weight. Statistical results for 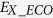 and 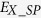 calculations cannot be affected, although systematic errors are yield as function of empirical values of dry weight.

### Gradient Boosting Machine and model evaluation

GBM analysis was performed on the key ecological indictors, namely E_X_ECO_ and E_X_SP_, using the variables filtered by Pearson correlation coefficients to avoid correlation levels higher than 0.7^24^. In our model we specified a Gaussian loss function with a 5-fold cross validation and the number of iterations set to 1500, an interaction depth of 5, and a learning rate of 0.005. A comprehensive introduction to this technique with parameterization is available^43^,^44^. The model was fitted using the R statistical package^45^. The Chla parameter was not shown in the list of independent covariates since the concentrations belonging to phytoplankton biomass were used to calculate E_X_ECO_ and E_X_SP_ indicators. The model performance was evaluated using four quantitative statistical methods: coefficient of determination (R^2^), Nash-Sutcliffe efficiency (NSE), ratio of the root-mean-square to the standard deviation of measured data (RSR), and percent bias (PBIAS), and the model prediction was judged as agreeable if NSE > 0.5, RSR 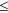 0.7 and PBIAS
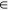
[−25%, 25%]^46^,^47^. The whole process of modeling was illustrated in Supplementary Material, Section 3.

## Results

### Field investigation of Taihu Lake

Table 1 shows the monitoring data statistics from this study. During the field investigation in 2013, there were 396 data points (33 sites × 12 months) for each measured variable. Inspection revealed that 92.4% of measured NO_2_-N concentrations were below the limit of detection (LOD) so these were not included in the GBM analysis. For the DTP and NH_4_-N variables which had low percentages of non-detects, the concentrations below LOD were substituted by one-half of the LOD. For NO_3_-N and PO_4_-P variables with approximately 23.0% and 38.9% of non-detects respectively, we randomly assigned a value to each non-detect using a normal distribution with one-half of the LOD as mean and one-fourth of the LOD as standard deviation. It indicated that 95% of the concentrations of interest (95% of the values fell within two standard deviations of the mean) ranged from 0 to the LOD. For any concentrations that were randomly assigned to be negative, we set them to zero, while the concentrations randomly assigned above the LOD were set to the LOD. The Chla, Phyt, Zoop, NH_4_-N, NO_3_-N and PO_4_-P variables had larger coefficients of variation (CV > 1), probably due to the high spatiotemporal heterogeneity in the lake.

From Table 2, there were five pairs of explanatory variables having strong correlations (R > 0.7, *p*-value < 0.05): WTEMP~DO, TN~NO_3_-N, TP~DTP, TP~PO_4_-P and DTP~PO_4_-P. In general, WTEMP is inversely related to DO, and dissolved inorganic nutrients are preferred to be taken up by planktonic algae and used for growth for physiological reasons^48^,^49^. The final set of filtered predicators that were applied in GBM analysis included NH_4_-N, NO_3_-N, PO_4_-P, WTEMP, TSS, PH, WIND, SOLR, WD, SDD and PREC. The simulation using TP instead of PO_4_-P was listed in Supplemental Material (Figs S4 and S5) for auxiliary analysis.

### Spatiotemporal distribution of eco-exergy and specific eco-exergy

**Figure 2:**
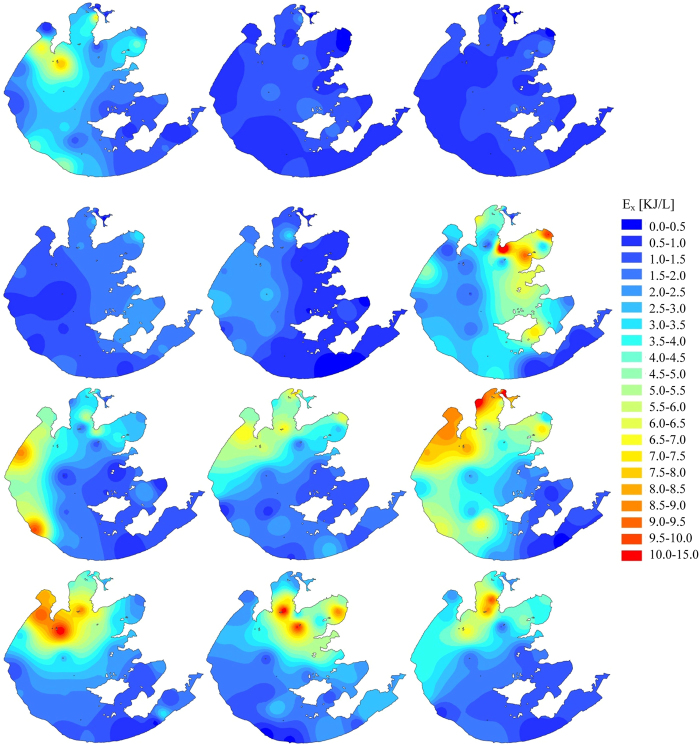
Spatiotemporal variation of eco-exergy of plankton community in Taihu Lake in 2013 (from top to bottom and from left to right: Winter - Dec, Jan, Feb; Spring - Mar, Apr, May; Summer - Jun, Jul, Aug; Fall - Sep, Oct, Nov).

**Figure 3:**
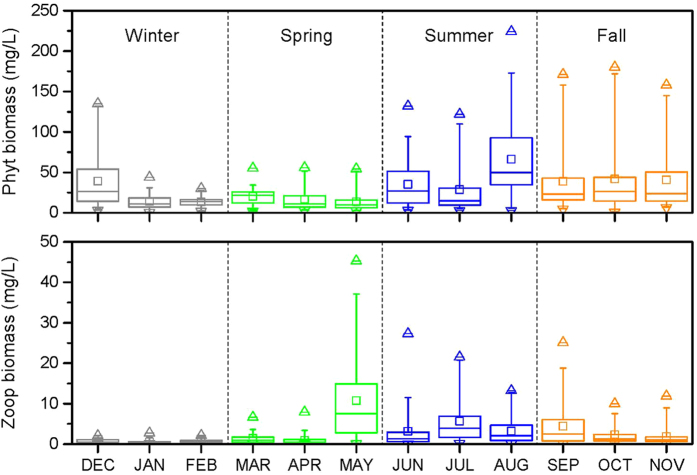
Time series of monthly observed phytoplankton and zooplankton biomass across 33 sampling sites in Taihu Lake in 2013 (Box and whisker represent 25^th^–75th and 5^th^–95th respectively; line within box represents median; quadrate within box represents mean; triangle and reverse triangle represent 1th and 99th respectively; upper and lower short strings represent maximum and minimum respectively).

The calculated eco-exergy and specific eco-exergy values on spatiotemporal distribution in Taihu Lake in 2013 were illustrated using Geographic Information System (GIS) interpolation using inverse distance weighted method. As shown in Fig. 2, the annual averages of the eco-exergy indicator in nine different sub-regions in the lake had followed the decreasing sequence: MB > ZB > GB > WZ > WB > CZ > EZ > SZ > DB, meaning that the total amount of biomass and information would accumulate in the northern part of the lake in which local primary production was potentially higher. Seasonal averages of the eco-exergy indicator in the lake were listed in descending order: summer, fall, spring and winter. The analysis revealed that the plankton community took on lower work energy of biomass and information during winter, with an average eco-exergy of 1.48 kJ/L. During spring, the plankton community grew and developed, showing a clear increase in eco-exergy, driving the system further from thermodynamic equilibrium. Further, storage of work energy in the lake greatly increased when quantities of work energy input from the solar radiation was utilized during summer and fall. Particularly in August, the system captured the maximum average eco-exergy of 4.79 kJ/L. During the entire year. In general, under optimal environmental conditions eco-exergy increases due to zooplankton growth from abundance of phytoplankton. However, the lake suffered from eutrophication due to the massive propagation of planktonic algae during the second half of 2013 (see Fig. 3). When the lake eutrophication emerged, we do have more biomass of phytoplankton for a time and the eco-exergy to measure biomass and information increased as well. Therefore, we cannot judge whether the aquatic ecosystem’s health is only based on the eco-exergy indicator.

**Figure 4:**
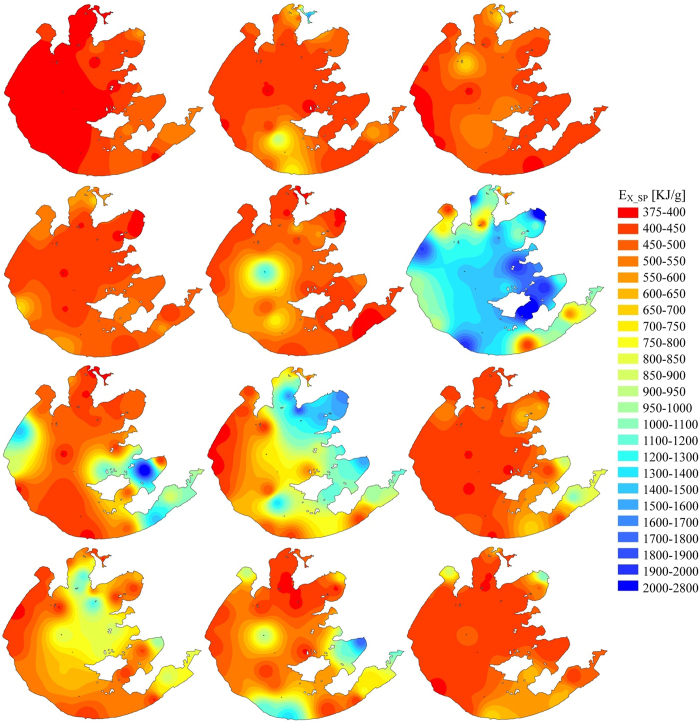
Spatiotemporal variation of specific eco-exergy of plankton community in Taihu Lake in 2013 (from top to bottom and from left to right: Winter - Dec, Jan, Feb; Spring - Mar, Apr, May; Summer - Jun, Jul, Aug; Fall - Sep, Oct, Nov).

**Figure 5:**
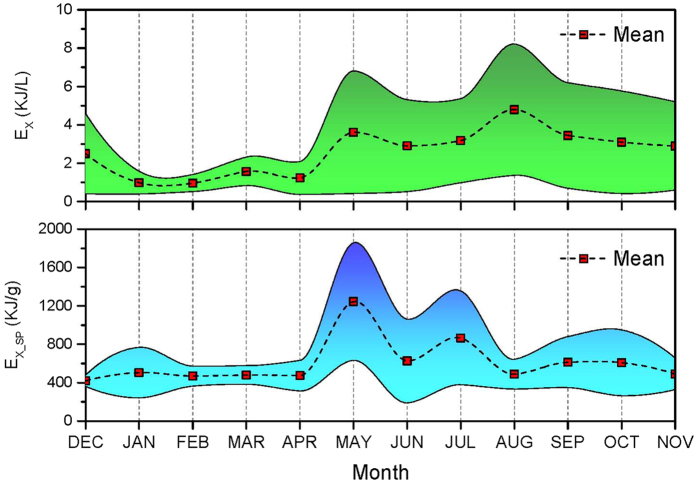
Calculated time series of eco-exergy and specific eco-exergy in Taihu Lake in 2013 (Shaded areas show 95% uncertainty intervals).

The specific eco-exergy indictor which expressed the dominance of the higher organisms carrying more information per unit of biomass was applied to assess lake ecosystem health^50^. The annual averages of the specific eco-exergy indicator in nine different sub-regions in the lake had followed the increasing sequence: ZB < WB < WZ < SZ < MB < CZ < DB < GB < EZ (see Fig. 4). Compared with eco-exergy values, Zhushan Bay had a great potential for eutrophication in that it exhibited a very high exergy but low specific eco-exergy, indicating that the biomass would be dominated by planktonic algae with low 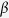-values. In general, Gonghu Bay presented a more healthy status as both eco-exergy (3.46 kJ/L) and specific eco-exergy (676.0 kJ/g) were relatively high in comparison with the other counterparts. As evidenced by both ecological indicators reaching the high level, as shown in Fig. 5, the lake remained healthy in May. From the time-varying trends, in August the plankton community with increasing eco-exergy but decreasing specific eco-exergy implied that the lake was prone to be eutrophic. It could also be demonstrated in Fig. 3 that phytoplankton biomass was in the highest level in August in 2013.

### Effects of abiotic factors on eco-exergy and specific eco-exergy

**Figure 6:**
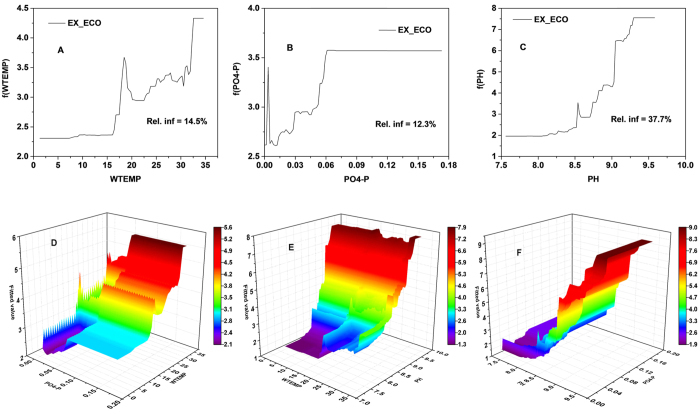
Single-variable and two-variable partial dependence plots for the three most influential predictor variables of eco-exergy in GBM model.

Both eco-exergy and specific eco-exergy were satisfactorily predicted by non-linear relationships with multiple abiotic factors. The statistical analysis resulted in values of 0.784 and 0.710 of R^2^, 0.77 and 0.69 of NES, 0.49 and 0.55 of RSR, and −8.7% and −4.6% of PBIAS for eco-exergy and specific eco-exergy, respectively (see Figs S2 and S3, Supplemental Material). For each ecological indicator, the top three variables having the highest relative influence together explained over 50% of the model variability. As shown in Fig. 6A–C, eco-exergy experienced a notable increase at water temperature higher than 16 °C, and a gradual increase over 20 °C. For dissolved inorganic nutrients, above the threshold of 1.75 μg/L of PO_4_-P, small increases in nutrient concentrations resulted in a dramatic increases in eco-exergy, followed by a plateau at higher concentrations. Increasing PH, in general, resulted in the elevated eco-exergy. Obviously, interaction analysis illustrated the interactive effect of any two-variable pair of WTEMP, PO_4_-P and PH on eco-exergy. Particularly, eco-exergy reached the highest level at moderate PH and PO_4_-P concentrations (see Fig. 6D–F).

**Figure 7:**
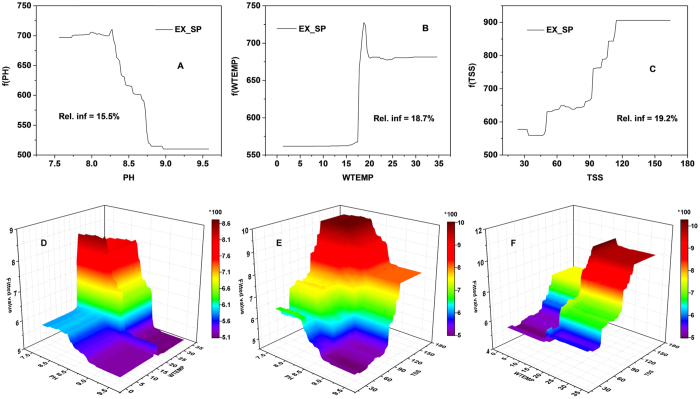
Single-variable and two-variable partial dependence plots for the three most influential predictor variables of specific eco-exergy in GBM model.

Specific eco-exergy was largely explained by PH (15.5%), WTEMP (18.7%) and TSS (19.2%) (see Fig. 7A–C). Slight increases in pH up to 8.2 predicted an almost linear decline in the specific eco-exergy. Again, specific eco-exergy increased gradually at low WTEMP, tipping at 17 °C and then started escalating, and finally kept steady above 20 °C. Specific eco-exergy was approximately directly proportional to TSS, however there was a flat relationship after TSS concentration reached 115 mg/L. The relationship between WTEMP and specific eco-exergy was weak at higher PH whereas decreased PH resulted in elevated specific eco-exergy at high WTEMP (Fig. 7D–F). At lower TSS and WTEMP (rising PH), the relationship between specific eco-exergy and the two variables were relatively inactive, however, elevated TSS and WTEMP (falling PH) was associated with increasing specific eco-exergy with two sharp thresholds. From Figs 6C and 7A, it was clearly shown that higher pH probably triggered propagation of planktonic algae.

## Discussion

This research investigated the performance of two ecological indicators, eco-exergy and specific eco-exergy and found both to be highly correlated with the biomass of plankton community. As shown in Fig. 3, during the whole year, both phytoplankton and zooplankton biomass varied from external perturbations and also from intrinsic prey-predator interactions in the aquatic community. For example, zooplankton levels were influenced not only by the amount of food (the phytoplankton), but also by the amount of predation by fish stock. In general, the total zooplankton community production rate increased along with phytoplankton production, indicating that both of them should increase in warmer months and decrease in winter. Meanwhile, the consumption rate also increased in warmer months, and therefore, the biomass does not exhibit a simple relationship. In this study, both phytoplankton and zooplankton biomass, in general, were at higher levels from spring through fall compared with significant decreases during the winter (see Fig. 3). The lake exhibited a classic spring bloom of zooplankton biomass followed by a decline in June, and then more steady decline of biomass through summer and fall even though phytoplankton concentrations stayed elevated, indicating that cyanobacteria harmful algal bloom (CyanoHAB) cannot be consumed effectively by zooplankton in Taihu Lake^51^. Meanwhile, a toxic prey might have destabilized the spatially homogeneity and resulted in high CV^52^. The main “outlier” was zooplankton biomass in May which was limited by the phytoplankton food supply. This suggested that phytoplankton biomass was probably suppressed in May due to high zooplankton grazing so that we could infer that the phytoplankton species shifted during the year. Some phytoplankton species would be easily grazed by zooplankton while others would be resistant to grazing. Also, from biomass observations zooplankton species shifted along with phytoplankton. This shows that the ecosystem has the capability to shift to the species better fitted to emerging conditions^53^. Our analysis also allowed us to judge whether structural changes occurred in the ecosystem by visualizing eco-exergy indicator variation^54^.

From the GBM analysis, we clearly see the two ecological indicators of plankton community in Taihu Lake are governed by complex and non-linear interactions with abiotic factors. The analysis also displays which conditions are favorable for the ecosystem: higher eco-exergy indicates more work energy in terms of biomass and information is stored in the ecosystem. The diagrams (see Figs 6 and 7) illustrated the total results of changed forcing functions and structural changes, as a result of the changed abiotic factors, e.g., water temperature, nutrient concentrations and total suspended solids. In general, those important abiotic factors positively impacted eco-exergy which is increased to its highest level and thereafter kept the same level. This result indicated that a shift from clear-water to turbid-water states probably occurred in the lake when PO_4_-P concentrations were increased from 0.03 to 0.06 mg/L, or 0.10 to 0.24 mg/L of TP (Figs S4 and S5, Supplemental Material) which is highly consistent with the investigations by Scheffer, *et al*.^3^ and Zhang, *et al*.^55^.

Eco-exergy is always increasing as a result of adaptation including changes in species composition because the ecosystem has a self-organizing ability to find the best solution under the prevailing conditions which is an expression of Darwin’s survival of the fittest^56^. However, the changing conditions (forcing functions) also cause structural changes, and definitely the eco-exergy. It was noteworthy that in this case it depicts that pH in the range of 7.5 to 8.2 (see Fig. 7D,E) provided the optimal environment for life, and then specific eco-exergy gradually decreased to the lowest level when pH reached 9.0. This implies that higher pH accompanied by eutrophication was even “killing” planktonic species^57^. Furthermore, the result was in accordance with field observations in surface waters of Mariager Fjord, Denmark in which pH of approximately 9.0 in July and August with phytoplankton blooms were observed^58^. We could also infer that elevated pH resulted from sufficient carbon taken up by phytoplankton, of which the growth was limited by inorganic carbon availability, e.g., CO_2_ or 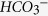 (see Figs 3, 5, 6C and 7A), and in such an eutrophic shallow lake, the rising CO_2_ level that would have shifted phytoplankton growth from carbon-limited to light-limited conditions likely intensified phytoplankton blooms^59^. The results indicated that we should pay more attention to climate change which is associated with increasing atmospheric CO_2_ levels, and could be a supplement to the N and P co-limitation management strategy for Taihu Lake^60^,^61^. Another interesting finding was the marginal effect of TSS in the water column on specific eco-exergy. Within certain concentration ranges more suspended solids (see Fig. 7C) was associated with a higher health level because organic matter could provide sufficient food available to detrivore zooplankton in the lake^62^.

Environmental measurements often include much variation (even sampling error) that cannot easily be explained in ecosystem health assessment so that the spatiotemporal sampling resolution needs to be improved. These sufficient observations can be also beneficial for revealing detailed mechanistic food web relationships and the bloom dynamics. For eco-exergy and specific eco-exergy calculations, we lumped groups of families together corresponding to available genomic information, e.g., algae species, instead of a specified single species, because until now too little information about the genomes are disclosed. The eco-exergy weighting factor *β* is based on dry weight, i.e. 18.7 kJ/g DW. We used empirical conversion factors for converting wet weight to dry weight in carbon unit. This systematic error could not affect the statistical results in this study, but for improving accuracy it will be necessary to obtained site-specific plankton dry weight biomass based on laboratory analysis. Furthermore, to better assess ecosystem health, benthos, macrophytes (the abundance of macrophytes is decreasing) and fish species in the lake should also be included.

Ecological systems may undergo catastrophic shifts, which abruptly shifts from one state to a contrasting one, across a tipping point as a result of changing external conditions, leading to substantial loss of ecosystem services^63^,^64^. Therefore, it is important to detect early-warning signals for critical transitions so that quick and effective management action can be implemented to prevent the “collapse”. On the one hand, based on eco-exergy theory and sufficient observations, we might judge the system behavior that might undergo development, succession and modification when exposed to variation in environmental conditions^65^,^66^. On the other hand, we also recommend that eco-exergy and specific eco-exergy indicators be applied to calculate statistical early warning signals such as variance, return rate and skewness to predict regime shifts from oligotrophic to hypertrophic state in Taihu Lake during the past six decades, using either long-term temporal or high-resolution spatial monitoring data^67^,^68^. Unfortunately, relevant research on anticipating critical transitions using these two ecological indicators has been scarce, possibly due to limited data availability. However, connecting eco-exergy theory with catastrophic theory opens up obvious new perspectives in the field of aquatic ecology, including ecosystem health assessments of shallow lakes worldwide.

## Additional Information

**How to cite this article:** Wang, C. *et al*. Effects of abiotic factors on ecosystem health of Taihu Lake, China based on eco-exergy theory. *Sci. Rep.* **7**, 42872; doi: 10.1038/srep42872 (2017).

**Publisher's note:** Springer Nature remains neutral with regard to jurisdictional claims in published maps and institutional affiliations.

## Supplementary Material

Supplementary Information

## Figures and Tables

**Table 1:** Statistics for measured variables across 33 sampling sites in Taihu Lake in 2013.

Variables	Chla	Phyt	Zoop	NH_4_-N^a^	NO_2_-N	NO_3_-N^b^	TN	PO_4_-P^b^	DTP^a^	TP	DO	TSS	pH	WTEMP	SDD	WD	WIND	SOLR	PREC
Min	2.10	0.67	0.058	0.013	<0.03	0	0.340	0	0.005	0.018	3.03	24.00	7.56	1.30	0.10	0.89	0	186.69	13.22
Mean	27.52	30.78	2.97	0.150	0.077	0.881	1.926	0.016	0.030	0.080	9.44	61.36	8.42	17.68	0.37	2.18	3.69	323.15	88.71
Max	238.00	224.00	45.30	2.070	0.210	4.070	7.060	0.173	0.190	0.448	13.07	164.00	9.58	34.60	1.18	2.97	9.20	472.67	178.51
SD	32.12	34.60	5.26	0.264	0.043	0.903	1.177	0.022	0.027	0.056	1.75	27.77	0.38	8.93	0.086	0.44	1.39	102.31	60.18
CV	1.17	1.12	1.77	1.76	0.562	1.03	0.61	1.38	0.90	0.70	0.19	0.45	0.05	0.51	0.23	0.20	0.38	0.32	0.68
P5	5.40	3.92	0.14	0.030	0.033	0.024	0.560	0.001	0.005	0.026	6.89	32.00	7.87	1.60	0.28	1.30	1.20	188.03	13.60
P50	15.80	17.95	1.00	0.070	0.067	0.637	1.700	0.007	0.021	0.066	9.20	53.00	8.38	18.35	0.37	2.27	3.70	323.33	92.06
P95	94.64	111.70	12.56	0.460	0.152	2.620	4.297	0.065	0.088	0.184	12.22	124.00	9.10	30.60	0.46	2.74	5.87	471.15	177.14
Samples	396	396	396	396	396	396	396	396	396	396	396	396	396	396	396	396	396	396	396
Non-detects	0	0	0	10	366	91	0	154	30	0	0	0	0	0	0	0	0	0	0

^a^Measurements below the LOD (NH_4_-N: 0.025 mg/L; NO_2_-N: 0.03 mg/L; NO_3_-N: 0.08 mg/L; PO_4_-P: 0.005 mg/L; DTP: 0.01 mg/L) were set to half to the LOD.

^b^Measurements below LOD were randomly assigned a value to each non-detect using a normal distribution with mean 1/2 the LOD and standard deviation 1/4 the LOD. The units of measured indicators are: Chla [mg/L]; Phyt [mg/L]; Zoop [mg/L]; NH_4_-N [mg/L]; NO_2_-N [mg/L]; NO_3_-N [mg/L]; TN [mg/L]; PO_4_-P [mg/L]; DTP [mg/L]; TP [mg/L]; DO [mg/L]; TSS [mg/L]; pH [dimensionless], WTEMP [°C]; SDD [m]; WD [m]; WIND [m/s]; SOLR [Langley]; PREC [mm].

**Table 2:** Pearson correlations for measured variables across 33 sampling sites in Taihu Lake in 2013.

Variables	PREC	WD	SDD	SOLR	PH	WTEMP	WIND	TN	TP	DO	DTP	PO_4_-P	NO_3_-N	NH_4_-N	TSS
PREC	1.00														
WD	0.07	1.00													
SDD	0.00	−0.13*	1.00												
SOLR	0.41*	−0.01	−0.08	1.00											
PH	0.10*	0.25*	0.05	0.12*	1.00										
WTEMP	0.60*	0.01	0.10*	0.69*	0.44*	1.00									
WIND	0.25*	0.20*	−0.03	−0.07	0.07	0.10	1.00								
TN	0.00	0.16*	−0.15*	0.20*	−0.18*	−0.20*	−0.02	1.00							
TP	0.19*	0.18*	−0.09	0.10*	0.33*	0.17*	0.00	0.39*	1.00						
DO	−0.57*	−0.01	−0.19*	−0.45*	−0.18*	−0.78*	−0.20*	0.14*	−0.12*	1.00					
DTP	0.09	0.16*	0.02	−0.06	0.04	−0.03	−0.01	0.54*	0.73*	−0.01	1.00				
PO_4_-P	0.08	0.16*	0.07	0.00	0.12*	0.06	−0.05	0.48*	0.74*	−0.10*	0.94*	1.00			
NO_3_-N	−0.09	0.12*	−0.15*	0.17*	−0.36*	−0.29*	−0.01	0.90*	0.08	0.15*	0.33*	0.27*	1.00		
NH_4_-N	−0.12*	0.03	0.00	−0.12*	−0.21*	−0.26*	−0.03	0.65*	0.29*	0.13*	0.57*	0.52*	0.50*	1.00	
TSS	0.07	0.04	−0.29*	0.09	−0.07	−0.08	0.22*	0.12*	0.19*	0.00	−0.07	−0.06	0.13*	−0.03	1.00

Two-tailed test of significance is used.

^*^Correlation is significant at the 0.05 level, calculated by OriginLab.
