# Supplementary material for: Effects of abiotic factors on ecosystem health of Taihu Lake, China based on eco-exergy theory
Source: Sci Rep. 2017 Feb 21;7:42872. doi: 10.1038/srep42872 (PMC5318890; doi:10.1038/srep42872)
Supplement: Supplementary Information [file srep42872-s1.pdf]

Supplemental Material

Effects of abiotic factors on ecosystem health of Taihu Lake, China based on eco-exergy theory

Ce Wang[a], Jun Bi[a,*], Brian D. Fath[b,c,*]

[a]State Key Laboratory of Pollution Control and Resource Reuse, School of the Environment, Nanjing University, Nanjing, 210023, P.R. China

[a,*]Corresponding Author

State Key Laboratory of Pollution Control and Resource Reuse

School of the Environment, Nanjing University

No. 163 Xianlin Avenue, Nanjing, 210023, P.R. China

Tel: 86-25-89681605; Fax: 86-25-89681605

E-mail: jbi@nju.edu.cn

[b,c,*]Corresponding Author

[b]Biology Department, Towson University, Towson, MD 21252, USA

[c]Advanced Systems Analysis Program, International Institute for Applied Systems Analysis (IIASA), Laxenburg, Austria

E-mail: bfath@towson.edu

1. Water smapling and sample pretreamnt

We carried on field observations at 33 different sites across the Taihu Lake at monthly intervals during the year of 2013. At each location, 4L of water sample was collected at a depth of 0.5m underneath water surface using organic glass collector. It was used to determine water quality variables, including NH_4-N, TN, NO_2-N, NO_3-N, TP, DTP, PO_4-P and Chl-a.

For observations of phytoplankton, 1L of water sample was collected at a depth of 0.5m using glass collector. 15mL of Lugol's solution served as fixative solution was added into the sample for preservation in case sample deterioration.

For observations of zooplankton, 1L of water sample was collected at a depth of 0.5m using glass collector. For quantifying microzooplantkon, water sample was input into 1L plastic flask with the addition of 10mL of Lugol's solution served as fixative solution. For quantifying macrozooplanton such as cladocerans and copepods, the sample was treated by 5% (v/v) formaldehyde solution.

Sampling, transportation and preservation of water samples were referred to [1,2]. All samples were kept in vehicle mounted refrigerator that was set to constant temperature of 4°C. Then, they are transported to laboratory within 4h.

2. Laboratory analysis of water samples

At each water sampling site, we used DO meter, pH microelectrode and secchi disk to measure DO concentration, water temperature, pH and water transparence of

the lake. We also used GPS to obtain the latitude and longitude of each location, and the anemoscope was used to determine wind speed and wind direction on the spot. All other variables were determined in laboratory.

2.1 Water quality indicators

The water sample was deposited for 30min, then supernatant extracted using Siphon method was used for TN and TP determinations. After this, the sample was filtered by acetic acid - nitric acid synthetic fabric membrane (47mm，0.45μm) and the filtrate was used for NH_4-N, NO_2-N, NO_3-N, DTP, PO_4-P. For Chl-a determination, the water sample was filtered by glass fiber membrane (47mm, 0.70μm), then filter membrane was put into 90% (v/v) acetone solution, and using solvent extraction it was kept for 4-12h. For TSS determination, the sample was filtered by oven-dried filter membrane (47mm, 0.45μm), then we dried the membrane again and weight it to calculate TSS mass. The detailed process, standard number and primary instrument during laboratory analysis were listed in Table S1.

Table S1 Chinese standards for laboratory analysis of water quality variables

WQ variable	Primary instrument	Standard number	Reference
NH_4-N	Cary50, UV spectrophotometer, Agilent, USA	HJ 535-2009	[3]
TN	Cary50, UV spectrophotometer, Agilent, USA	GB 11894-89	[4]
NO_2-N	ICS-90 ion chromatography, Metrohm, Swizerland	SL 86-1994	[5]

NO_3-N	ICS-90 ion chromatography, Metrohm, Swizerland	SL 86-1994	5
PO_4-P	Cary50, UV spectrophotometer, Agilent, USA	GB 11893-89	6
DTP	Cary50, UV spectrophotometer, Agilent, USA	GB 11893-89	6
TP	Cary50, UV spectrophotometer, Agilent, USA	GB 11893-89	6
TSS	M2010, analytical balance, Mettler Toledo, USA	GB 11901-89	7
Chl-a	Cary50, UV spectrophotometer, Agilent, USA	SL 88-1994	8
DO	YSI-550 DO meter, YSI, USA	HJ 506-2009	9
WTEMP	YSI-550 DO meter, YSI, USA	GB 13195-1991	10
pH	pH330, YSI, USA	GB 6920-86	11
SDD	SD-20 Secchi disc, Purity, China	SL 87-1994	12

2.2 Phytoplankton biomass

Microscopic counting method was used for phytoplankton determination. We took the well-shook water sample (⩾100 ml) after treated by Lugol’s solution, then carried on vacuum filtration using suction filter equipped with cellulose acetate membrane (45mm,1.2μm). The membrane with algae cells was put into beaker and then we added 5-8mL of purified water. After mixed, extracted solution was preserved.

Repeat the process for 3-5 times and the final volume of lotion was fixed to 30mL.

The solution was deposited and shook up, then we extracted 0.1mL and injected it into counting box ($20\times20mm^2$). When covering the cover slip, it was warranted that there was no bubble inside the counting box, and no spill. The rapid detection count method was used by microscopic examination 10 × 40, 400X. The phytoplankton biomass expressed in mg/L was obtained by unit conversion in relation to number of phytoplankton cells per liter.

2.3 Zooplankton biomass

Microscopic counting method was used for zooplankton determination. For microzooplantkon determination, 1000mL of water sample was standing for 24h, and siphon pipe covered #25 plankton net was used to take up supernatant fraction. The remaining 10-30mL deposit was transferred into 50mL volumetric flask and then supernatant fraction was removed by siphon pipe and leave 10mL of deposit solution to be determined. concentrated into 10mL.

Generally, we collected macrozooplanton species, such as cladocerans and copepods, using #13 plankton net, and put it into 50mL volumetric flask. After standing for 24h, supernatant fraction was removed by siphon pipe and leave 10mL of deposit solution to be determined. When counting number, the deposit solution should be shook up, then using micro pipette we extracted 0.1mL and injected it into 0.1mL of counting box (20mm×20mm). The rapid detection count method was used by microscopic examination 10×40, 400X for counting protozoan, rotifer and nauplius.

For counting cladoceran and copepod, using micro pipette we extracted 1.0mL and injected it into 1.0mL of counting box (40mm×60mm). The microscopic examination 10×10, 100X was used. The zooplankton biomass expressed in mg/L was obtained by unit conversion in relation to number of individual zooplankton per liter.

2.4 Wet weight to C-biomass conversion

Approximately, 0.16 (the average of 0.22, 0.16, 0.11 and 0.16) was used to convert phytoplankton wet biomass to dry weight biomass in carbon unit[13]. For zooplankton species, we obtained that the ratio of dry weight to wet weight of biomass was approximately 0.19[14], and carbon biomass roughly 32% of zooplankton dry weight[15]. Therefore, the unit conversion was 0.06 for converting wet weight to carbon biomass[16].

3. Statistical method

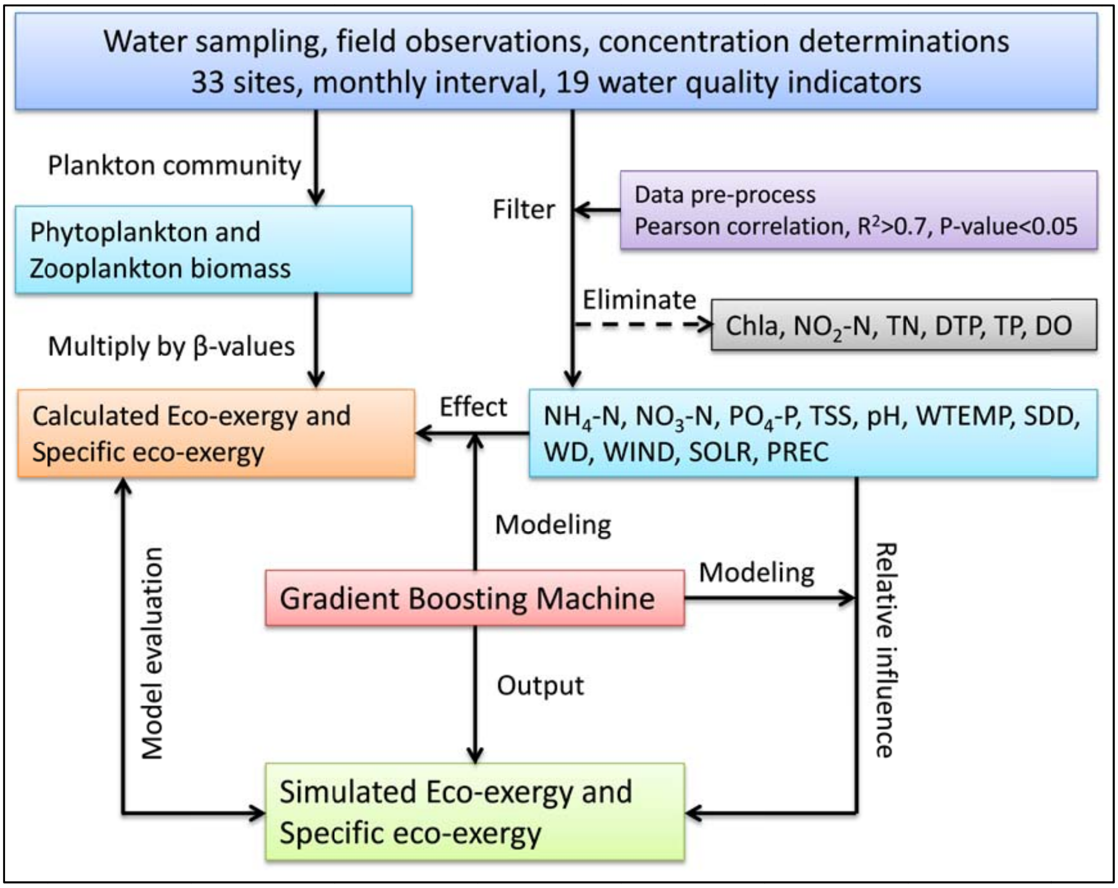

Fig.S1 Flow chart of evaluating the effects of abiotic factors on ecosystem health of Taihu Lake using GBM

In 2013, we carried out field investigations at monthly intervals at 33 different sampling sites in Taihu Lake, and collected a large amount of observed data, including phytoplankton biomass, zooplankton biomass, chlorophyll-a, NH_4-N, NO_2-N, NO_3-N, TN, PO_4-P, DTP, TP, DO, TSS, pH, WTEMP, SDD, WD, WIND, SOLR, PREC. All indicators were determined synchronously for each sampling time and site. Those data of phytoplankton and zooplankton biomass were used for calculating eco-exergy and specific eco-exergy indicators. After filtered by data pre-process and variable correlation, for GBM modeling we finally obtained two response variables -

eco-exergy and specific eco-exergy, and eleven predicator variables - NH_4-N, NO_3-N, PO_4-P, TSS, pH, WTEMP, SDD, WD, WIND, SOLR, PREC.

GBM is a decision-tree based approach, and thus highly non-linear without having to transform the independent variables. We used the package “gbm” in R-language, and then outputting a file of predictions. The model was a forest of tiny decision trees that come together to form a solution. The significant advantage of GBM was to provide a convenient and computationally efficient way to explore highly non-linear interactions between independent variables and the dependent variable. We implemented the linear fit of calculated values of ecological indicator of interest versus simulated results derived from GBM to evaluate model performance. We could also determine what independent variables were having the most impact on the solution space, from looking at the “influence” of each independent variable. The resulting relative influence of each independent variable basically shows what percentage of the choices in all the decision trees that make up the final solution rely on each independent variable.

4. GBM results

Fig.S2 showed that the linear fit of calculated eco-exergy and predicted eco-exergy from GBM in which calculated eco-exergy indicator was considered as dependent variable, and NH_4-N, NO_3-N, PO_4-P, TSS, pH, WTEMP, SDD, WD, WIND, SOLR, PREC indicators were considered as independent variables.

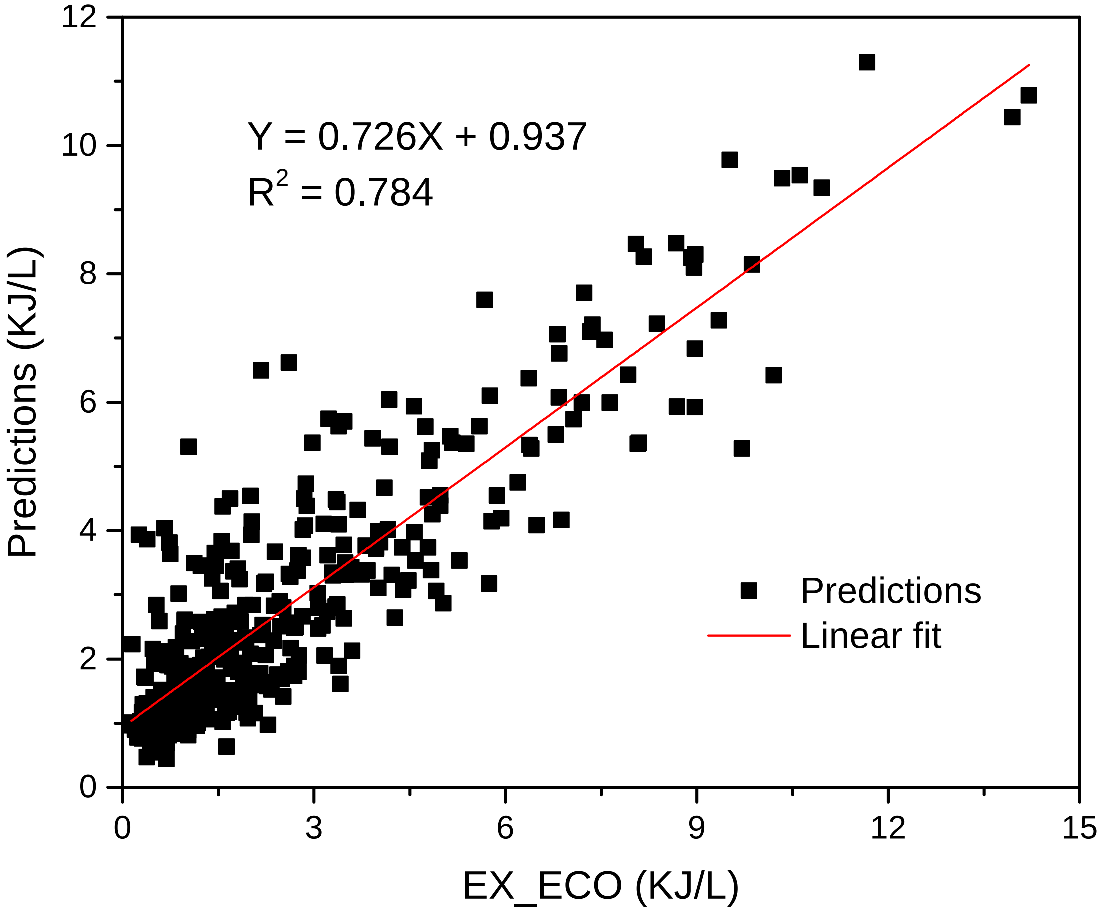

Fig.S2 Linear fit of predicted eco-exergy values from GBM and calculated ones obtained from observations (PO_4-P is used as one of covariates).

Fig.S3 showed that the linear fit of calculated specific eco-exergy and predicted specific eco-exergy from GBM in which calculated specific eco-exergy indicator was considered as dependent variable, and NH_4-N, NO_3-N, PO_4-P, TSS, pH, WTEMP, SDD, WD, WIND, SOLR, PREC indicators were considered as independent variables.

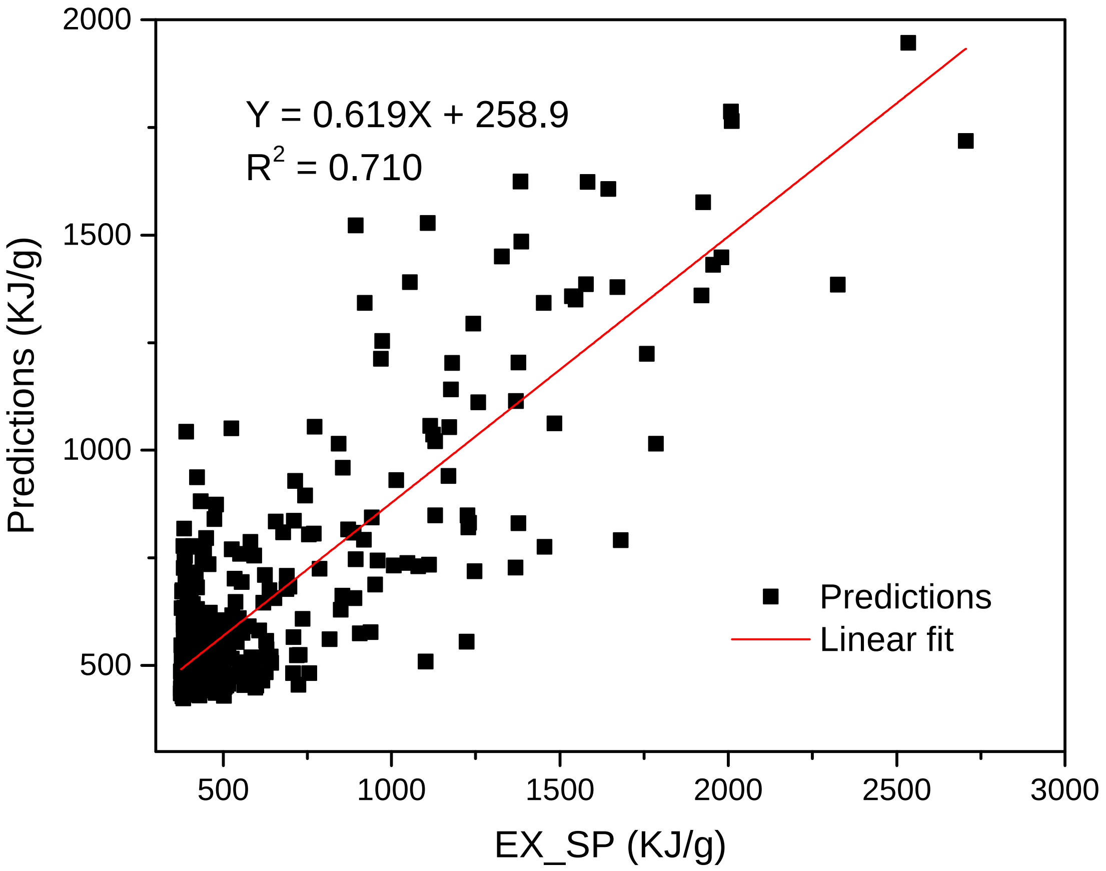

Fig.S3 Linear fit of predicted specific eco-exergy values from GBM and calculated ones obtained from observations (PO_4-P is used as one of covariates).

Fig.S4 showed that the linear fit of calculated eco-exergy and predicted eco-exergy from GBM in which calculated eco-exergy indicator was considered as dependent variable, and NH_4-N, NO_3-N, TP, TSS, pH, WTEMP, SDD, WD, WIND, SOLR, PREC indicators were considered as independent variables.

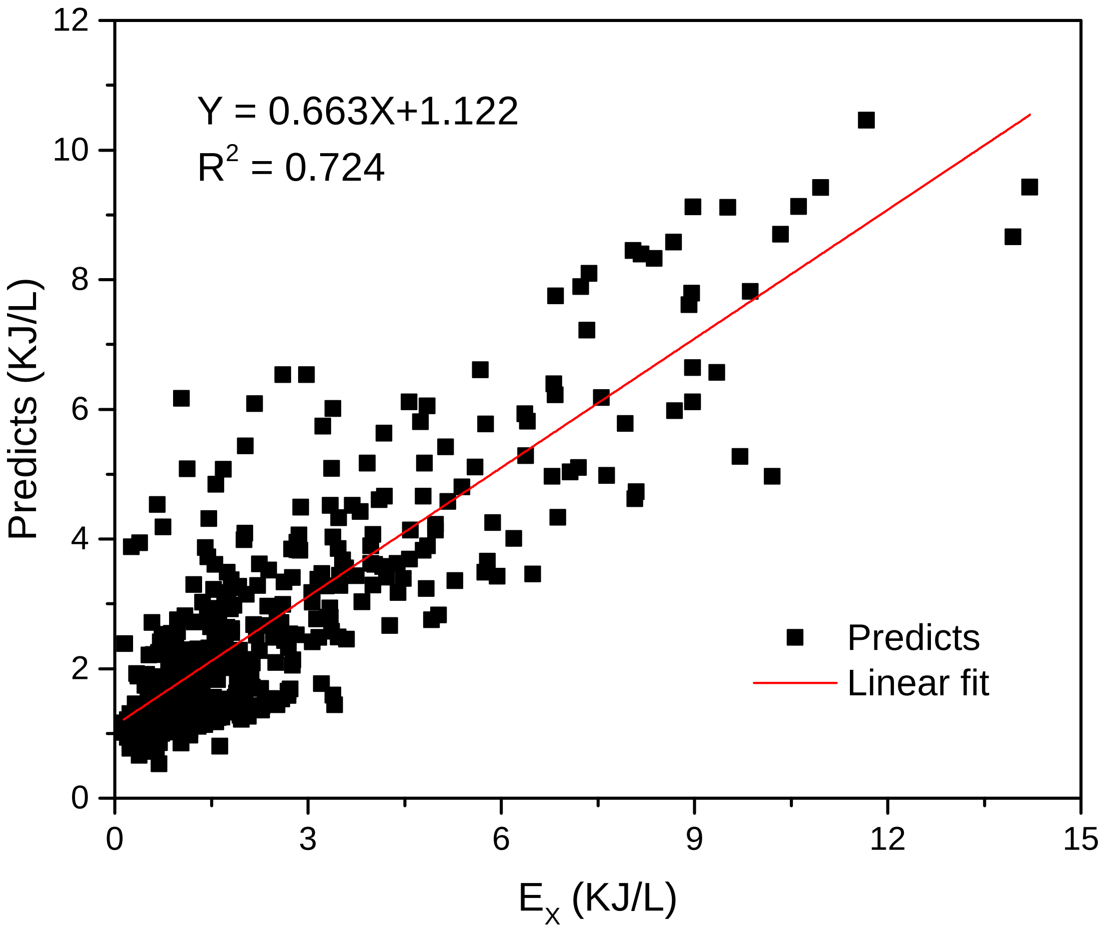

Fig.S4 Linear fit of predicted eco-exergy values from GBM and calculated ones obtained from observations (TP is used as one of covariates).

Fig.S5 showed that single-variable partial dependence plots for the TP predictor variable of eco-exergy in GBM in which calculated eco-exergy indicator was considered as dependent variable, and NH_4-N, NO_3-N, TP, TSS, pH, WTEMP, SDD, WD, WIND, SOLR, PREC indicators were considered as independent variables.

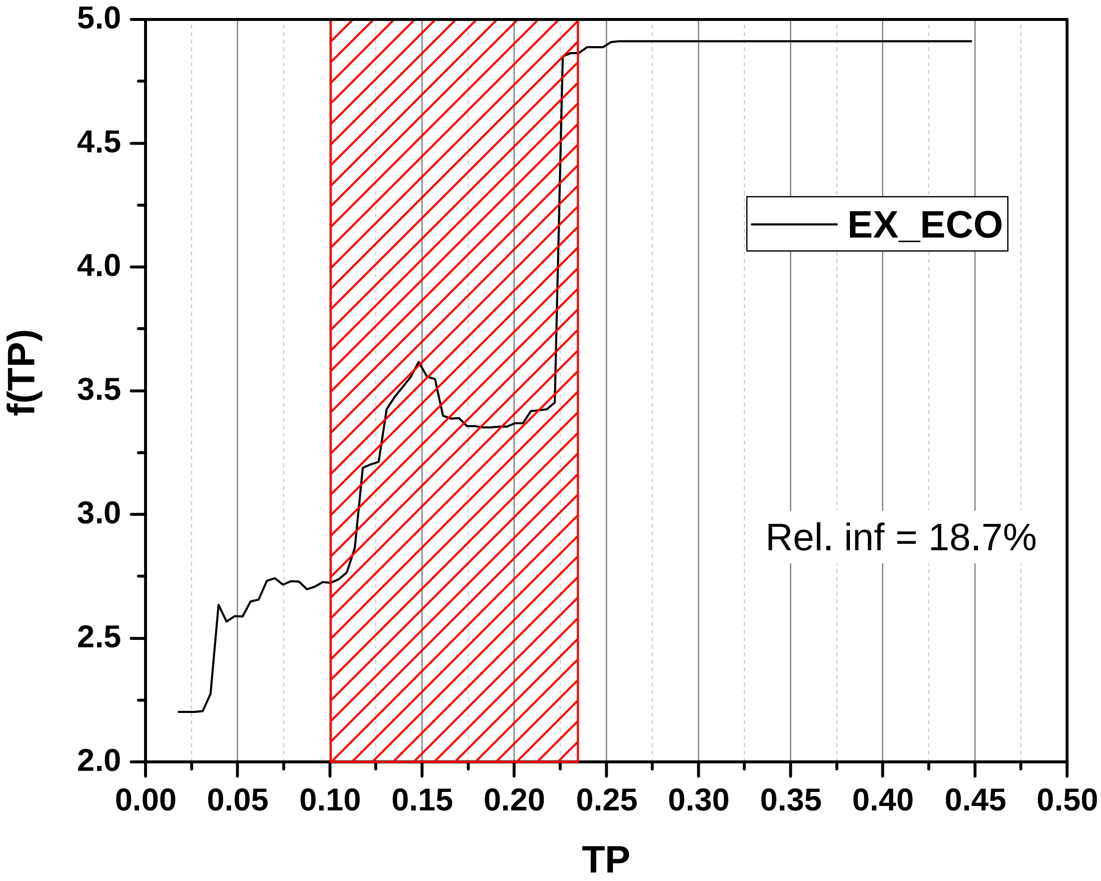

Fig.S5 Single-variable partial dependence plots for the TP predictor variable of eco-exergy in GBM model (Relative influence is approximately 18.7%).

5. Data of plankton biomass

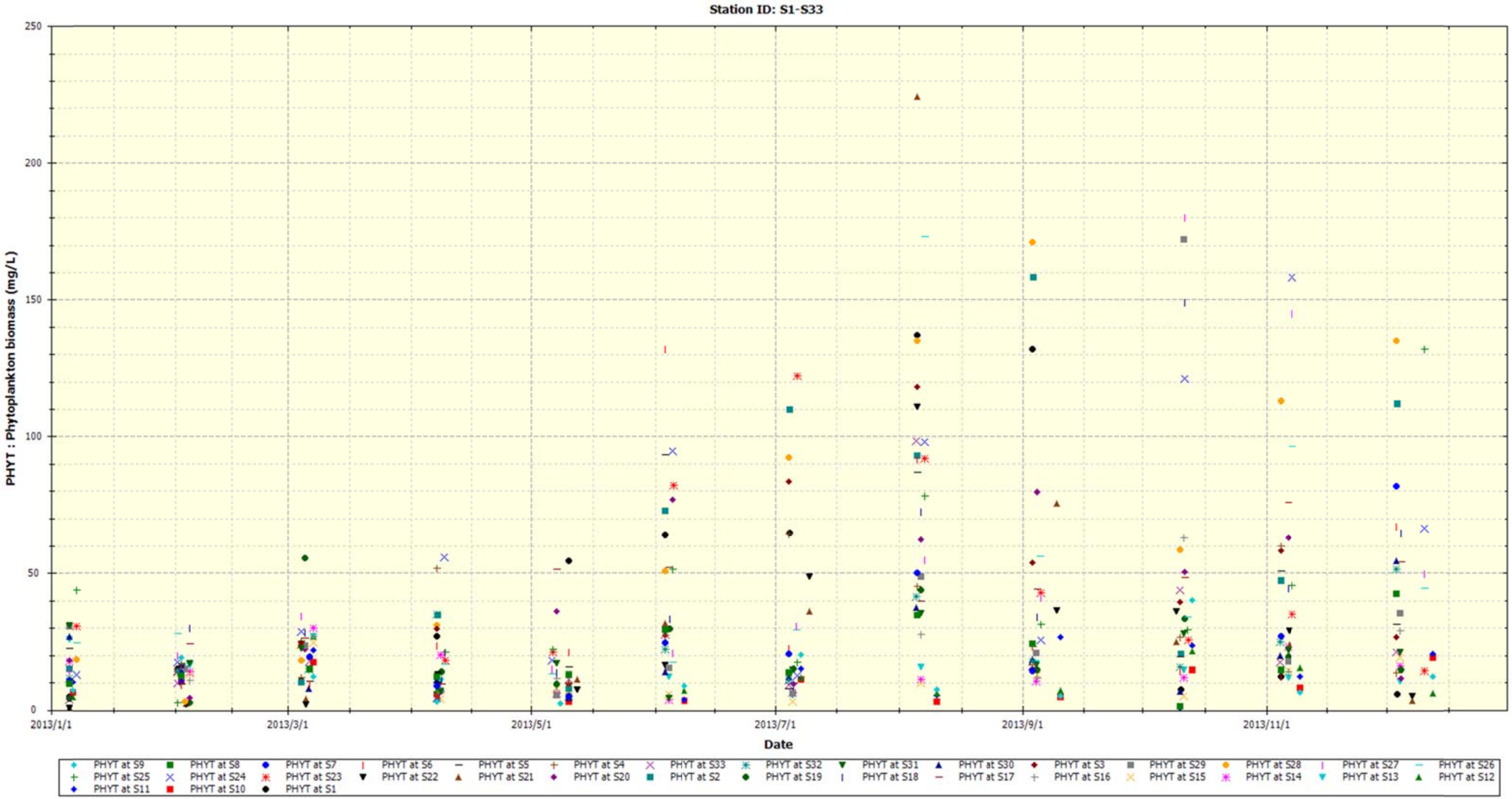

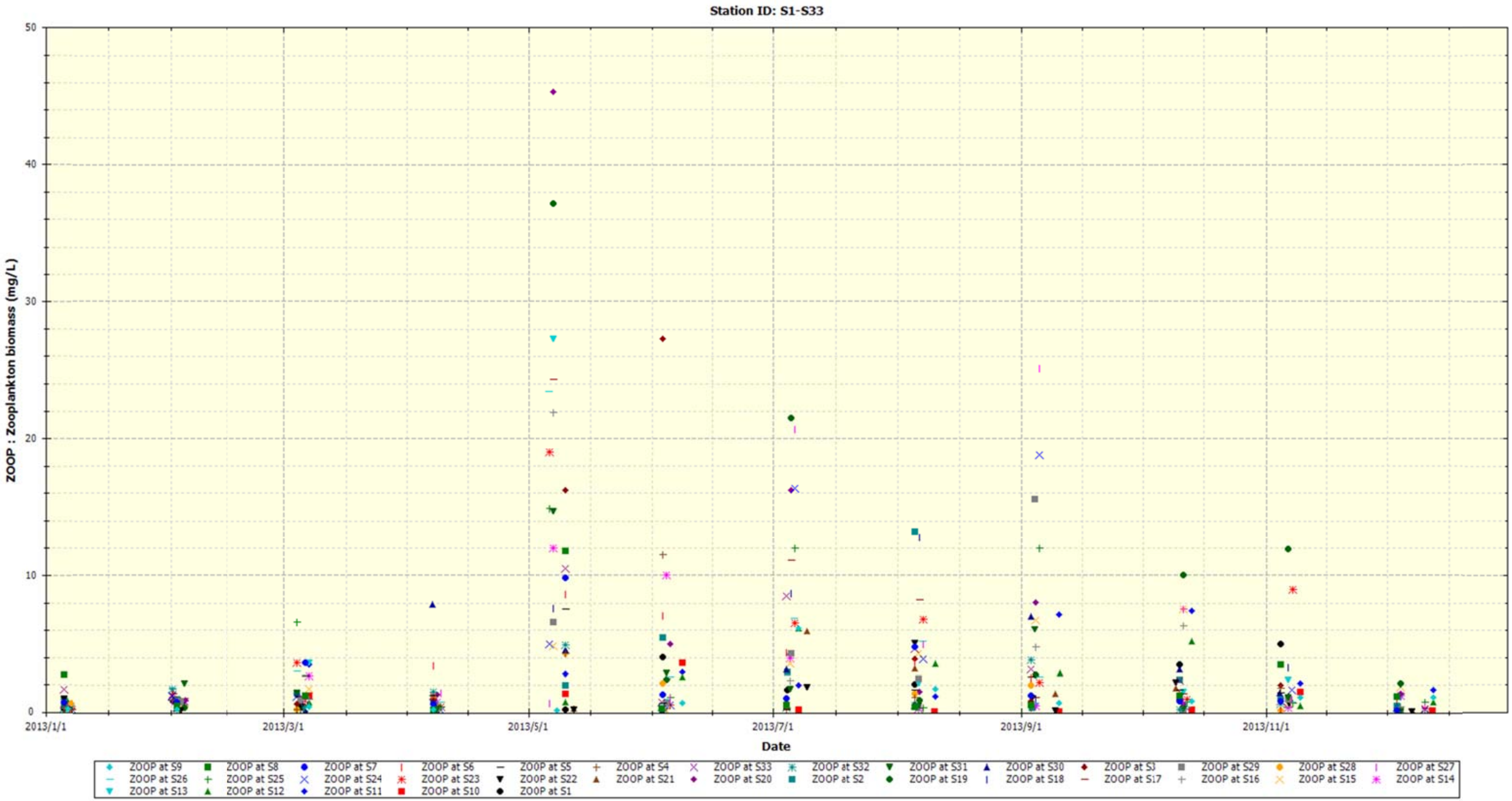

Station ID: S1-S33
ZOOP : Zooplankton biomass (mg/L)
0
10
20
30
40
50
2013/1/1
2013/3/1
2013/5/1
2013/7/1
2013/9/1
2013/11/1
Date
ZOOP at S9
ZOOP at S8
ZOOP at S7
ZOOP at S6
ZOOP at S5
ZOOP at S4
ZOOP at S33
ZOOP at S32
ZOOP at S31
ZOOP at S30
ZOOP at S3
ZOOP at S29
ZOOP at S28
ZOOP at S27
ZOOP at S26
ZOOP at S25
ZOOP at S24
ZOOP at S23
ZOOP at S22
ZOOP at S21
ZOOP at S20
ZOOP at S2
ZOOP at S19
ZOOP at S18
ZOOP at S17
ZOOP at S16
ZOOP at S15
ZOOP at S14
ZOOP at S13
ZOOP at S12
ZOOP at S11
ZOOP at S10
ZOOP at S1

References

1. MWR, Regulation for water environmental monitoring. In The Ministry of Water Resources of the People's Republic of China: Beijing, 2013; Vol. SL 219-2013.
2. *Standards assembly of water conservancy techniques*. China WaterPower Press: Beijing, 2002.
3. MEP, Water quality-Determination of ammonia nitrogen-Nessler's reagent spectrophotometry. In Ministry of Environmental Protection of the People's Republic of China: Beijing, 2009; Vol. HJ 535-2009.
4. MEP, Water quality-Determination of total nitrogen-Alkaline potassium persulfate digestion-UV spectrophotometric method. In Ministry of Environmental Protection of the People's Republic of China: Beijing, 1989; Vol. GB 11894-89.
5. MWR, Determination of inorganic anions in water (Ion chromatography method). In The Ministry of Water Resources of the People's Republic of China: Beijing, 1994; Vol. SL 86-1994.
6. MEP, Water quality-Determination of total phosphorus-Ammonium molybdate spectrophotometric method. In Ministry of Environmental Protection of the People's Republic of China: Beijing, 1989; Vol. GB 11893-89.
7. MEP, Water quality-Determination of suspended substance-Gravimetric method In Ministry of Environmental Protection of the People's Republic of China: Beijing, 1989; Vol. GB 11901-89.
8. MWR, Determination of Chlorophyll (Spectrophotometric method). In The Ministry of Water Resources of the People's Republic of China: Beijing, 1994; Vol. SL 88-1994.
9. MEP, Water quality-Determination of dissolved oxygen-Electrochemical probe method. In Ministry of Environmental Protection of the People's Republic of China: Beijing, 2009; Vol. HJ 506-2009.
10. MEP, Water quality-Determination of water temperature-Thermometer or reversing thermometer method. In Ministry of Environmental Protection of the People's Republic of China: Beijing, 1991; Vol. GB 13195-91.
11. MEP, Water quality-Determination of pH value-Glass electrode method. In Ministry of Environmental Protection of the People's Republic of China: Beijing, 1986; Vol. GB 6920-86.
12. MWR, Determination of transparency (Diaphanometer and disc method). In The Ministry of Water Resources of the People's Republic of China: Beijing, 1994; Vol. SL 87-1994.
13. Yacobi, Y. Z.; Zohary, T., Carbon:chlorophyll a ratio, assimilation numbers and turnover times of Lake Kinneret phytoplankton. *Hydrobiologia* **2009,** *639*, (1), 185-196.
14. Madin, L. P.; Horgan, E. F.; Steinberg, D. K., Zooplankton at the Bermuda Atlantic Time-series Study (BATS) station: diel, seasonal and interannual variation in biomass, 1994–1998. *Deep Sea Research Part II: Topical Studies in Oceanography* **2001,** *48*, (8), 2063-2082.
15. Wiebe, P. H.; Boyd, S.; Cox, J. L., Relationships between zooplankton displacement volume, wet weight, dry weight, and carbon. *Available from the National Technical Information Service, Springfield VA 22161 as ADA-022 723, Price codes: A 02 in paper copy, A 01 in microfiche. Fishery Bulletin* **1975,** *73*, (4), 777-786.
16. Yamaguchi, A.; Watanabe, Y.; Ishida, H.; Harimoto, T.; Maeda, M.; Ishizaka, J.; Ikeda, T.; Mac Takahashi, M., Biomass and chemical composition of net-plankton down to greater depths (0–5800m)

in the western North Pacific Ocean. *Deep Sea Research Part I: Oceanographic Research Papers* **2005,** *52*, (2), 341-353.
